# Multimorbidity and its association with health-related quality of life among older adults in india: a cross-sectional analysis of LASI wave-1

**DOI:** 10.1186/s12877-025-06427-1

**Published:** 2025-09-29

**Authors:** Vasim Ahamad, Raza Mohammad, Anil Kumar Pal, K. R. Chouhan

**Affiliations:** 1 Department of Migration and Urban Studies, International Institute for Population Sciences, Mumbai, 400088 India; 2 Department of Public Health and Mortality Studies, International Institute for Population Sciences, Mumbai, 400088 India; 3 Department of Population and Development, International Institute for Population Sciences, Mumbai, 400088 India; 4 Department of Geography, University of Allahabad, Prayagraj, 211002 India

**Keywords:** Multimorbidity, HRQoL, EQ-5D, Older adults, India

## Abstract

**Background:**

Multimorbidity is common among older people and is associated with a reduction in quality of life, including physical and psychological dimensions of health. This study aims to examine the association between multimorbidity and health-related quality of life (HRQoL) by gender and key socioeconomic factors among older adults in India.

**Method:**

The Longitudinal Ageing Study in India (LASI) Wave-I data were used, and older persons aged 60 and above were selected for the study, which included 30,716 final samples. The HRQoL was measured based on a EuroQol Five-Dimension (EQ-5D) measure. The preliminary study used descriptive statistics to examine the baseline characteristics of the sample, the prevalence of chronic conditions, and the mean EQ-5D score. Aside from that, the findings on the association of EQ-5D score with exposure and the independent variables were carved out using a multiple linear regression model. Furthermore, the results were stratified by gender and tested for interactions.

**Results:**

This study revealed that 23.8% of older adults had multimorbidity, with a higher prevalence among females and older age groups. Hypertension (32.7%), bone/joint diseases (19.6%), diabetes (14.3%), and stroke (2.5%) were common conditions. Individuals with multimorbidity exhibited significantly lower HRQoL scores (mean: 10.53) compared to those with single (8.98) or no morbidity (7.54). Adjusted regression models confirmed that multimorbidity (β = 2.19, 95%CI: 2.04,2.35) and female gender (β = 0.86, 95%CI: 0.71,1.00) strongly predicted poorer HRQoL. The association between multimorbidity and HRQoL was stronger in females compared to males. Lower socioeconomic status, rural residence, and poor self-rated health further exacerbated these disparities. These findings highlight the negative impact of multimorbidity on the physical and psychological well-being of older adults in India.

**Conclusions:**

This study found that multimorbidity significantly reduces HRQoL among older adults in India, with women, individuals of lower socioeconomic status, and those reporting poor self-rated health experiencing the most significant burden. These findings highlight the need for gender-sensitive, equity-focused public health strategies aimed at managing chronic conditions and improving quality of life among the ageing population in India.

## Background

Chronic diseases have increased continuously in recent decades worldwide, affecting people from all walks of life and all ages, and they have devastating health consequences for individuals, families, and communities [1]. According to the latest World Health Organisation (WHO) report, four major chronic diseases collectively killed about 35 million people in 2021. These major chronic diseases are cardiovascular disease (19 million), cancer (10 million), chronic respiratory disease (4 million), and diabetes (2 million) [1]. Chronic conditions lead to adverse health outcomes such as disability, poor quality of life, and mortality [2]. Preventing and controlling these diseases is a significant development challenge for the 21st century, especially in the low- and middle-income countries (LMICs).

LMICs such as India are facing ageing due to increased life expectancy and reduced fertility over the decades [3]. According to the 2011 census, 103 million people (8.6%) were aged 60 and above [4]. Nonetheless, the older population is expected to account for 19.1% of the overall population by 2050, with 319 million individuals aged 60 and above [5]. With rising longevity, multimorbidity (the coexistence of two or more chronic diseases) and comorbidities have become progressively common for the older population [6, 7].

Findings from the current Longitudinal Ageing Study in India (LASI) suggest that 18% of the individuals aged 45 years and above were affected with Multimorbidity, 28% of the individuals aged 45 and above were suffering from cardiovascular diseases (CVDs), 12% had diabetes, 6% had chronic lung disease, and about 9% were suffering from arthritis in 2018 [8–10].

The multimorbidity condition significantly varies with gender characteristics, as several previous studies found that the prevalence of multimorbidity was higher among women than men [10–14]. These disparities between men and women were in the number of chronic ailments and the cluster of specific diseases [15–17]. This disparity has been attributed to biological, sociocultural, economic, or environmental causes, which signifies the importance of gender [18–21].

The presence of multimorbidity among older individuals is significantly associated with negative well-being and poor quality of life. Multiple studies, including recent research in India and international systematic reviews, have demonstrated that older adults with multimorbidity experience lower scores in various domains of quality of life, including physical, psychological, and social well-being [12, 13, 21–26]. The negative impact is consistent across community, outpatient, and hospital settings, highlighting the importance of targeted interventions to address the complex needs of this population [27].

The health-related quality of life (HRQoL) is a comprehensive concept encompassing positive and negative aspects of an individual’s life and includes subjective evaluations of their physical, social, emotional, psychological, and mental functioning [28, 29]. It goes beyond direct measures of population health, life expectancy, and causes of death and focuses on the impact of health status on quality of life. In contemporary public health practice, HRQoL has emerged among the most essential measurable outcomes of health programs and interventions in the last few decades. It has been seen as a reliable measure that can account for the actual improvement in patients’ and the general population’s overall health status [28]. The multimorbidity condition increases mortality risk and functional decline, negatively impacting health-related quality of life (HRQoL) [30]. In India, where there is an increase in the prevalence of multimorbidity [31]. Residents with multimorbidity have not received enough attention regarding their HRQoL. However, some studies have shown that multimorbidity significantly negatively affects HRQoL in India [32, 33].

Multimorbidity is associated with substantial adverse outcomes, including functional decline, higher healthcare utilization, and diminished HRQoL. Several studies have shown that multimorbidity adversely affects HRQoL, particularly in older adults. However, much of the Indian evidence is based on small-scale or region-specific datasets with limited focus on gender and socioeconomic disparities. While a few studies highlighted the negative impact of multimorbidity on HRQoL [11, 32], there remains a lack of nationally representative research examining these associations in detail. Moreover, gender differences significantly influence the burden of multimorbidity and HRQoL. Older women in India often experience higher rates of chronic conditions and lower quality of life due to biological, social, and economic disparities [11, 21, 34]. Stratifying the analysis by gender allows us to capture these differences and provides more targeted insights for gender-sensitive public health interventions.

Addressing this gap, the present study uses data from the Longitudinal Ageing Study in India (LASI) to examine the association between multimorbidity and HRQoL among older adults in India, and to assess how this relationship varies by gender and key socioeconomic factors.

## Method and materials

### Data source

This study was based on cross-sectional survey data. The data for the analysis were drawn from the Longitudinal Ageing Study in India (LASI) wave-1, 2017–18. It is a nationally representative survey of 73,396 individuals, 31,135 male and 42,261 aged 45 and older, and their spouses (regardless of age) across all states and union territories of India. The survey’s main objective was to study the health status and socioeconomic well-being of older adults in India. The LASI adopted a multistage stratified area probability cluster sampling design to arrive at the eventual observation units: older adults aged 45 and above and their spouses, irrespective of age [8].

The present study was conducted on respondents aged 60 years and above. After excluding individuals aged less than 60 years (41,494) and those with missing data (1,186), the final sample size comprised 30,716 older adults.

### Study variables

#### Outcome variable

The health-related quality of life (HRQoL) was the primary outcome variable of the study. HRQoL represents a multidimensional construct that captures individuals’ perceptions of how health conditions affect their physical, mental, and social functioning [35, 36]. HRQoL provides valuable insights into patient-centred outcomes beyond traditional clinical measures. The EuroQol Five-Dimension (EQ-5D) instrument has emerged as a standardized approach to quantify HRQoL across diverse populations and health conditions, focusing specifically on dimensions influenced by health status [37].

The EQ-5D framework examines five distinct health dimensions: mobility, self-care, usual activities, pain/discomfort, and anxiety/depression. This instrument has evolved over time, with versions featuring different levels of response categories - namely the EQ-5D-3 L (three levels) and the EQ-5D-5 L (five levels), where “L” indicates the number of response levels within each dimension [37].

In this study, the EQ-5D was adapted to a binary response format (yes/no) for each dimension due to the structure of the LASI dataset. Each dimension was assessed through multiple related questions (except pain/discomfort, which used a single item), and the anxiety/depression domain incorporated the Center for Epidemiological Studies-Depression (CES-D) scale for a more nuanced assessment. Scores across all five dimensions were aggregated to create a composite HRQoL index, with higher scores indicating poorer quality of life. The full methodological details and operationalization of HRQoL in this context have been described in previous research [25].

The comprehensive HRQoL index resulted in a theoretical range of 0 to 33 (observed mean = 8.681). This composite demonstrated strong internal consistency (scale reliability coefficient = 0.84). Higher values indicate greater health-related difficulties (poorer HRQoL), while lower scores reflect fewer functional limitations (better HRQoL).

#### Exposure variable

Multimorbidity constitutes the primary exposure variable in this study. The LASI dataset provided information on nine physician-diagnosed chronic conditions, assessed through self-reported measures confirmed by healthcare professionals. Participants were explicitly asked: *“Has any health professional ever diagnosed/told you that you have the following chronic conditions or diseases?”*

The examined conditions included: elevated cholesterol levels, chronic lung diseases, chronic cardiac conditions, stroke, hypertension, diabetes mellitus, cancer/malignant tumours, musculoskeletal disorders (bone/joint diseases), and neurological/psychiatric conditions. Additional information regarding the diagnosing physician, diagnosis timeline, and current treatment status was collected for affirmative responses. Chronic illness status was dichotomized as “No” (absence of diagnosed chronic conditions) or “Yes” (presence of at least one diagnosed chronic condition from the nine categories).

#### Independent variables

The analysis incorporated multiple sociodemographic and behavioural factors as independent variables based on the previous studies [25]. These included age (stratified as 60–69, 70–79, and 80 + years), biological sex (male/female), residential setting (rural/urban), marital status (currently married, widowed, or other), religion (Hindu, Muslim, or other), education (none, primary, secondary/higher, or graduate and above), Monthly Per Capita Consumption Expenditure (MPCE) quintile (categorized as poorest, poorer, middle, richer, and richest), employment status (currently working, not working, or never worked), tobacco use (yes/no), alcohol consumption (yes/no), physical activity engagement (yes/no), and self-perceived health status (good/poor).

#### Statistical analysis

The preliminary study used descriptive statistics to examine the baseline characteristics of the sample, the prevalence of chronic conditions, and the mean EQ-5D score. Aside from that, the findings on the association of EQ-5D score with exposure and the independent variables were carved out using a multiple linear regression model. The linear regression model is expressed as follows:

$$\mathrm Y\;=\;{\mathrm\beta}_0\;+\;{\mathrm\beta}_1{\mathrm x}_1\;+\;{\mathrm\beta}_2{\mathrm x}_2\;+\;...\;+\;{\mathrm\beta}_{\mathrm k}{\mathrm x}_{\mathrm k}\;+\;\mathrm\varepsilon$$

Where *Y* represents the outcome variable, $\beta_0$ is the intercept, and $X_1,\;X_2,\;X_3,\;....\;,\;X_k$ was the set of explanatory variables, $\beta_1\;\beta_2\;\beta_1\;........\;\beta_k$ were the regression coefficients to be estimated in the model, and $\varepsilon$ was the error term [38].

For the robustness of the model, we have checked the model linearity, multicollinearity, and goodness of model fit. We found that the model linearly fit as an insignificant *p-value*, and we also found the goodness of the model fit in all models. Furthermore, variance inflation factors (VIF < 10) confirmed the absence of multicollinearity, and residual analyses validated linearity and homoscedasticity assumptions. The proper individual-level sampling weights were used to make the results representative. Moreover, we tested the interaction term between Multimorbidity and gender. The statistical package STATA for Windows version 16 was used for all statistical analyses.

## Results

### Background characteristics of the respondents

The mean age of the study participants was 68.7 years (standard deviation: 7.4). Table 1 depicts the respondents’ baseline characteristics stratified by sex. The weighted result shows that approximately 60% of respondents were aged 60–69 among both male and female respondents. The majority of the respondents were rural residents (71.1%), currently married (61.8%), and had no formal education (56.4%). A higher proportion of currently married, higher education is shown among males than females. The other backward class (45.2%) and the Hindu religion (82.7%) showed a higher proportion in caste and religious characteristics, and in this proportion, no variation was found between males and females. Only 31.5% of respondents worked during the survey, and 16.4% had the richest Monthly Per Capita Consumption Expenditure (MPCE) quintile. Among males, the proportion of working status was higher than that of females. Approximately 89% lived with family members, 5.69% lived alone, and 24% belonged to the East region. 40.16% of respondents smoked ever, 14.7% drank alcohol, and approximately 27% did physical activity. Among smoking, alcohol, and physical activity, the proportion of males is higher than that of females. The self-rated health characteristics showed that 48.7% had poor condition. Approximately 49% reported poor health conditions among respondents; females reported more poor health conditions than males.

**Table 1:** Baseline socio-demographic and health characteristics of study samples by sex

Characteristics	Male	Female	Total
	*N* (%)	*N* (%)	*N* (%)
Age			
60–69	8566 (59.12)	9719 (59.9)	18,285 (59.53)
70–79	4375 (30.19)	4769 (29.39)	9143 (29.77)
80+	1550 (10.69)	1738 (10.71)	3288 (10.7)
Residence			
Rural	10,568 (72.93)	11,270 (69.46)	21,838 (71.1)
Urban	3922 (27.07)	4956 (30.54)	8878 (28.9)
Marital status			
Currently married	11,755 (81.12)	7229 (44.55)	18,984 (61.8)
Widowed	2391 (16.5)	8690 (53.56)	11,081 (36.08)
Others	344 (2.38)	307 (1.89)	651 (2.12)
Education			
No-education	5567 (38.42)	11,770 (72.54)	17,337 (56.44)
Primary	4316 (29.78)	2686 (16.55)	7002 (22.8)
Secondary and higher secondary	3579 (24.7)	1536 (9.47)	5116 (16.65)
Graduate and above	1029 (7.1)	233 (1.43)	1261 (4.11)
Caste category			
Scheduled caste	2773 (19.13)	3087 (19.02)	5859 (19.08)
Scheduled tribes	1126 (7.77)	1363 (8.4)	2489 (8.1)
Other backward class	6589 (45.47)	7295 (44.96)	13,884 (45.2)
Others	4003 (27.63)	4481 (27.62)	8484 (27.62)
Religion			
Hindu	12,002 (82.83)	13,414 (82.67)	25,416 (82.74)
Muslim	1572 (10.85)	1710 (10.54)	3282 (10.68)
Others	917 (6.33)	1102 (6.79)	2019 (6.57)
Working status			
Working	6527 (45.04)	3140 (19.35)	9666 (31.47)
Not working	7964 (54.96)	13,086 (80.65)	21,050 (68.53)
MPCE quantile			
Poorest	3038 (20.97)	3667 (22.6)	6705 (21.83)
Poorer	3150 (21.74)	3543 (21.83)	6693 (21.79)
Middle	3054 (21.07)	3325 (20.49)	6378 (20.77)
Richer	2794 (19.28)	3103 (19.13)	5897 (19.2)
Richest	2454 (16.94)	2588 (15.95)	5043 (16.42)
Living Arrangement			
Living with family	13,606 (93.9)	13,720 (84.56)	27,326 (88.96)
Living with others	528 (3.65)	1113 (6.86)	1642 (5.34)
Living alone	356 (2.46)	1392 (8.58)	1749 (5.69)
Regions			
North	1825 (12.6)	2107 (12.98)	3932 (12.8)
Central	3286 (22.68)	3147 (19.4)	6433 (20.94)
East	3603 (24.87)	3727 (22.97)	7331 (23.87)
West	2368 (16.34)	2932 (18.07)	5300 (17.26)
South	2975 (20.53)	3816 (23.52)	6791 (22.11)
Northeast	433 (2.99)	497 (3.06)	929 (3.02)
Ever smoked			
No	5785 (39.92)	12,595 (77.63)	18,380 (59.84)
Yes	8705 (60.08)	3630 (22.37)	12,336 (40.16)
Ever alcohol			
No	10,386 (71.67)	15,803 (97.4)	26,189 (85.26)
Yes	4105 (28.33)	422 (2.6)	4527 (14.74)
Physical activity			
No	9296 (64.15)	13,270 (81.78)	22,565 (73.46)
Yes	5195 (35.85)	2956 (18.22)	8151 (26.54)
Self-rated health			
Good	7698 (53.12)	8058 (49.66)	15,756 (51.3)
Poor	6792 (46.88)	8168 (50.34)	14,960 (48.7)
Total	**14,490 (100)**	**16,226 (100)**	**30,716 (100)**

### Prevalence of multimorbidity among older adults

**Fig. 1:**
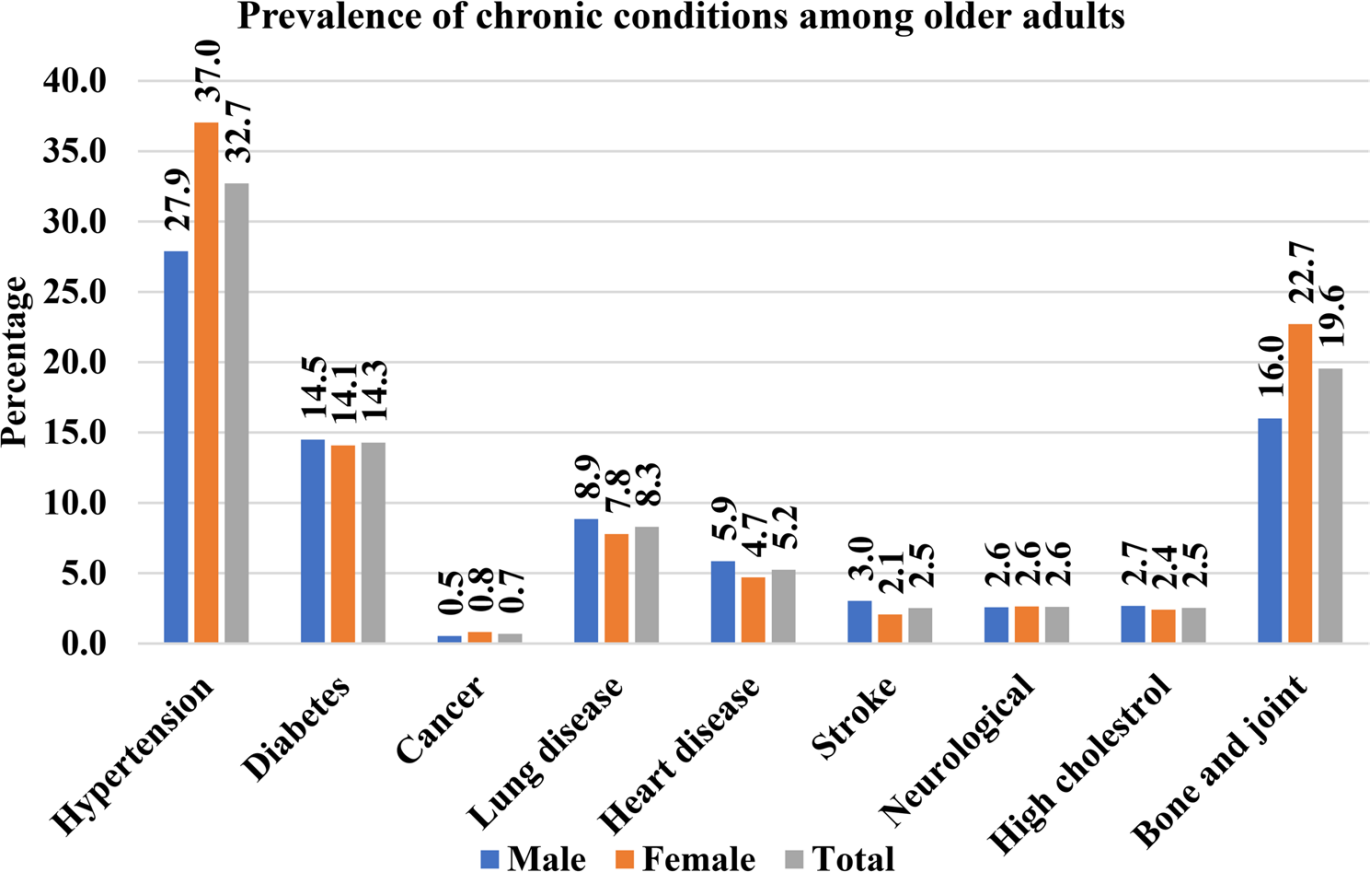
Prevalence of Chronic Conditions among Older Adults (≥ 60 Years) by sex in India

Figure 1 shows the prevalence of nine selected chronic disease conditions among older adults by sex. Hypertension [32.7% (95%CI: 32.71–32.73)], bone/joint diseases [19.6% (95%CI: 19.53–19.56)], diabetes [14.3% (95%CI: 14.27–14.29)], lung disease [8.3% (95%CI: 8.28–8.31)], and heart disease [5.2% (95%CI: 5.24–5.25)] were the five most prevalent chronic diseases among older adults in India. Cancer [0.7% (95%CI: 0.68–0.70)], stroke [2.5% (95%CI: 2.51–2.52)], high cholesterol [2.5% (95%CI: 2.53–2.54)], and neurology [2.6% (95%CI: 2.60–2.61)] were the least prevalent among older adults. Chronic conditions such as hypertension [37% (95%CI: 37.03–37.06) vs. 27.9% (95%CI: 27.86–27.89)], bone/joint diseases [22.7% (95%CI: 22.69–22.71) vs. 16% (95%CI: 16.00-16.02)], and cancer [0.8% (95%CI: 0.81–0.82) vs. 0.5% (95%CI: 0.54–0.55)] were more prevalent among females than males. In contrast, females were less prevalent than males in some chronic cases such as Diabetes (14.1% (95%CI: 14.08–14.1) vs. 14.5% (95%CI: 14.49–14.5)], lung disease [7.8% (95%CI: 7.79–7.80) vs. 8.9% (95%CI: 8.84–8.86)], heart disease [4.7% (95%CI: 4.70–4.71) vs. 5.9% (95%CI: 5.85–5.86)], high cholesterol [2.4% (95%CI: 2.40–2.41) vs. 2.7% (95%CI: 2.68–2.69)], and stroke [2.1% (95%CI: 2.05–2.06) vs. 3.0% (95%CI: 3.03–3.04)].

**Fig. 2:**
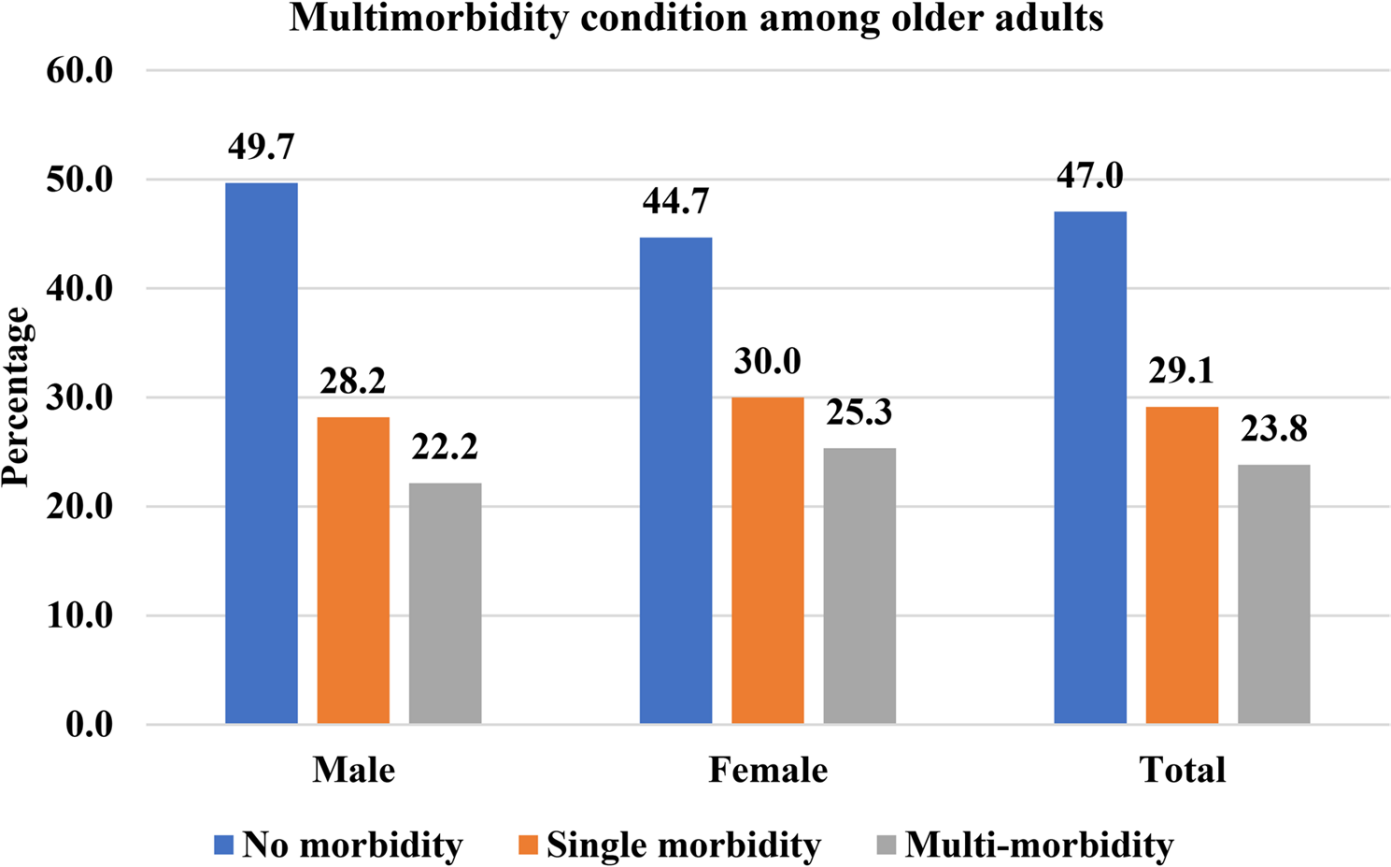
Prevalence of Single and Multimorbidity among Older Adults by Sex in India

Figure 2 shows the prevalence of Multimorbidity among older adults by sex. 23.8% of the total had Multimorbidity, whereas approximately 29% had single morbidity. Among males, approximately 50.3% had one or more chronic conditions. In this, 28.2% had single conditions and 22.2% had multimorbidity conditions, respectively. The prevalence of chronic conditions among females was higher (55.3%). In this, 30% had single morbidity, and 25.3% had multimorbidity conditions. The multimorbidity prevalence was higher among females than males.

### Health-related quality of life (HRQoL) with the chronic conditions

**Fig. 3:**
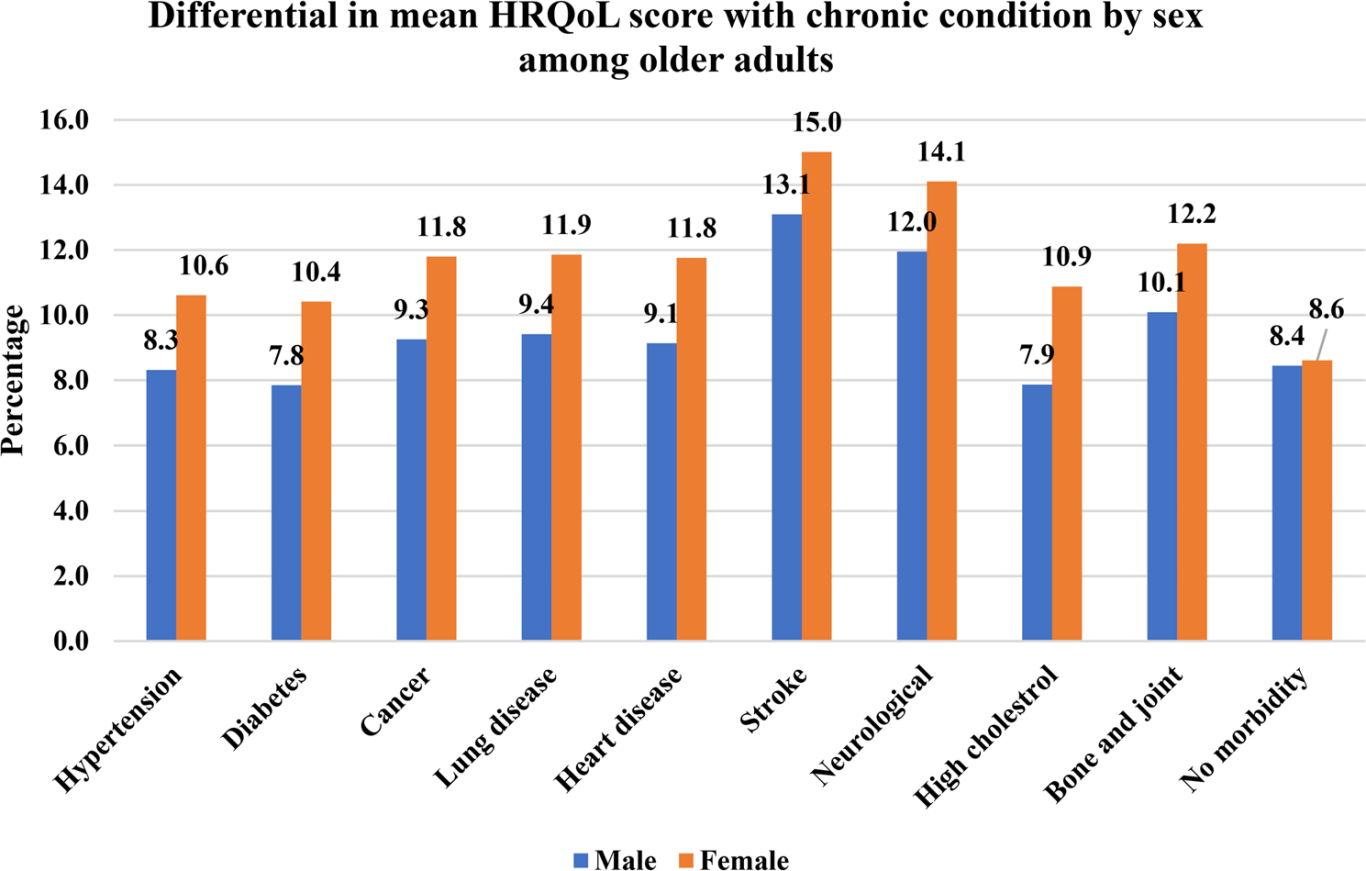
Differential in mean HRQoL scores with chronic condition by sex among older adults in India

Figure 3 shows the HRQoL score with chronic conditions stratified by sex. The HRQoL score was represented in mean value, and a higher value shows a lower HRQoL. The figure shows that females had higher HRQoL scores (8.6) than males (8.4). Further, the figure shows that females with chronic conditions had higher scores of HRQoL than males. The HRQoL shows lower with a chronic condition such as stroke (13.1 vs. 15), neurological (12 vs. 14.1), bone/joint diseases (10.1 vs. 12.2), lung disease (9.4 vs. 11.9), heart disease (9.1 vs. 11.8), and in these conditions, males had lower HRQoL than females, respectively.

Table 2 depicts the HRQoL mean value with background characteristics stratified by sex. The HRQoL score with multimorbidity status was higher (10.53) than single (8.98) and no morbidity (7.54); that indicates the individual with multimorbidity condition significantly had lower HRQoL, and it also varies with gender characteristics as results show the males had a value of HRQoL score (9.25) with multimorbidity than the females (11.59). The HRQoL score increases with factors such as higher age, rural residence, widowed status, no education, not working, poorest wealth, living alone, and poor health status, which indicates these characteristics were related to lower HRQoL in later life, and similar results are shown among male and female individuals.

**Table 2:** Mean HRQoL score across Socio-demographic and health factors among older adults by sex in India

Indicators	Male	Female	Total	*p*-value
	mean (SD)	mean (SD)	mean (SD)	
Morbidity status				
No morbidity	6.48 (4.99)	8.61 (5.78)	7.54 (5.50)	< 0.001
Single morbidity	7.79 (5.64)	10 (5.95)	8.98 (5.91)	< 0.001
Multimorbidity	9.25 (6.29)	11.59 (6.32)	10.53 (6.41)	< 0.001
Age				
60–69	6.41 (4.91)	8.47 (5.33)	7.5 (5.24)	< 0.001
70–79	8.34 (5.76)	11.19 (6.26)	9.76 (6.18)	< 0.001
80+	11.46 (6.76)	14.17 (7.03)	12.88 (7.03)	< 0.001
Residence				
Rural	7.79 (5.65)	10.06 (6.13)	8.96 (6.01)	< 0.001
Urban	6.9 (5.49)	9.34 (6.00)	8.2 (5.89)	< 0.001
Marital status				
currently married	7.19 (5.42)	8.58 (5.52)	7.72 (5.50)	< 0.001
widowed	9.03 (6.18)	10.96 (6.38)	10.55 (6.39)	< 0.001
others	8.54 (6.40)	9.45 (5.89)	9 (6.16)	< 0.001
Education				
No-education	8.54 (5.96)	10.28 (6.25)	9.72 (6.21)	< 0.001
Primary	7.66 (5.50)	9.14 (5.68)	8.26 (5.62)	< 0.001
Secondary and higher secondary	6.43 (5.14)	8.22 (5.35)	6.93 (5.26)	< 0.001
Graduate and above	5.55 (4.70)	7.1 (5.20)	5.9 (4.86)	< 0.001
Caste category				
Scheduled caste	7.87 (5.75)	10.1 (6.18)	9.03 (6.08)	< 0.001
Scheduled tribes	6.87 (5.34)	8.64 (5.90)	7.8 (5.71)	< 0.001
Other backward class	7.77 (5.68)	10.04 (6.06)	8.94 (5.99)	< 0.001
Others	7.29 (5.56)	10.03 (6.12)	8.72 (6.02)	< 0.001
Religion				
Hindu	7.59 (5.62)	9.95 (6.10)	8.82 (5.99)	< 0.001
Muslim	7.88 (5.72)	10.81 (6.26)	9.39 (6.18)	< 0.001
Others	6.75 (5.42)	8.38 (5.71)	7.6 (5.63)	< 0.001
Working status				
Working	5.86 (4.12)	7.58 (4.76)	6.41 (4.41)	< 0.001
Not working	8.7 (6.23)	10.31 (6.25)	9.67 (6.29)	< 0.001
MPCE quantile				
Poorest	7.91 (5.72)	10.16 (6.33)	9.11 (6.15)	< 0.001
Poorer	7.65 (5.62)	9.73 (6.02)	8.74 (5.92)	< 0.001
Middle	7.34 (5.61)	9.73 (6.03)	8.59 (5.95)	< 0.001
Richer	7.33 (5.45)	9.78 (5.96)	8.59 (5.84)	< 0.001
Richest	7.25 (5.64)	9.61 (6.12)	8.44 (6.00)	< 0.001
Living Arrangement				
Living with family	7.43 (5.57)	9.62 (6.03)	8.52 (5.91)	< 0.001
Living with others	8.49 (5.89)	11.25 (6.51)	10.4 (6.45)	< 0.001
Living alone	8.83 (6.43)	10.66 (6.21)	10.25 (6.30)	< 0.001
Regions				
North	6.94 (5.56)	9.23 (6.04)	8.13 (5.92)	< 0.001
Central	7.95 (5.70)	10.27 (6.15)	9.1 (6.04)	< 0.001
East	8.22 (5.73)	10.69 (6.05)	9.47 (6.02)	< 0.001
West	7.05 (5.60)	9.63 (6.20)	8.46 (6.07)	< 0.001
South	7.9 (5.70)	10.35 (6.16)	9.2 (6.08)	< 0.001
Northeast	6.51 (5.01)	8.17 (5.51)	7.36 (5.34)	< 0.001
Ever smoked				
No	7.18 (5.65)	9.69 (6.10)	8.82 (6.06)	< 0.001
Yes	7.75 (5.57)	10.22 (6.07)	8.51 (5.84)	< 0.001
Ever alcohol				
No	7.75 (5.57)	10.22 (6.07)	8.51 (5.84)	< 0.001
Yes	7.32 (5.37)	9.77 (5.90)	7.63 (5.50)	< 0.001
Physical activity				
No	8.26 (5.98)	10.29 (6.19)	9.42 (6.19)	< 0.001
Yes	6.05 (4.48)	7.77 (5.18)	6.7 (4.83)	< 0.001
Self-rated health				
Good	5.77 (4.40)	7.77 (5.16)	6.76 (4.89)	< 0.001
Poor	9.77 (6.19)	11.95 (6.27)	10.97 (6.33)	< 0.001
Total	**7.5 (5.61)**	**9.81 (6.10)**	**8.7 (5.98)**	**< 0.001**

*SD* Standard Deviation

### Association between multimorbidity and HRQoL

Table 3 depicts the association of influencing Multimorbidity and other factors with HRQoL among older adults in India. The results were presented in adjusted coefficients with 95% CI. The analysis in model-1 for total indicated that the adjusted β coefficients were 2.19 (β: 2.19; 95%CI: 2.04,2.35; P: <0.001) and 0.92 (β: 0.92; 95%CI: 0.79,1.06; P: <0.001) for persons with multimorbidity and single morbidity, respectively, compared to no morbidity; this result indicates that the participants with morbidity condition significantly had lower level of HRQoL. In model-1, the adjusted β coefficients were 0.86 (β: 0.86; 95%CI: 0.71,1.00; P: <0.001) for female older adults concerning males, which indicates that females had significantly lower HRQoL than males. The adjusted β coefficients were shown to be significantly positive with increased age, widowed status, not working status, regions, and poor health status, concerning their reference counterparts, and the adjusted β coefficients were shown to be significantly negative with higher education, richest wealth, engage in physical activity, concerning their reference category. There were no significant associations between β coefficients in the caste category and living arrangement characteristics. Approximately similar results were shown in model-2 for males and model-3 for females in the association of multimorbidity on HRQoL. The interaction term between being female and having multimorbidity conditions was statistically significant (β = 0.34, 95% CI: 0.05, 0.62; *p* = 0.020), indicating that the association between multimorbidity conditions and HRQoL was stronger among females than males. However, the interaction for a single chronic condition was not statistically significant (β = 0.20, CI: −0.07 to 0.47; P: 0.149).

**Table 3:** Adjusted regression results for the associations of HRQoL with Multimorbidity

HRQoL Score	Model-1 (Total)	Model-2 (Male)	Model-3 (Female)
	β [95% CI]	β [95% CI]	β [95% CI]
Morbidity status			
No morbidity^®^	0 [0.00,0.00]	0 [0.00,0.00]	0 [0.00,0.00]
Single morbidity	0.92*** [0.79,1.06]	0.85*** [0.66,1.04]	0.99*** [0.80,1.19]
Multimorbidity	2.19*** [2.04,2.35]	2.07*** [1.85,2.28]	2.29*** [2.07,2.52]
Age			
60–69^®^	0 [0.00,0.00]	0 [0.00,0.00]	0 [0.00,0.00]
70–79	1.37*** [1.24,1.51]	0.96*** [0.78,1.14]	1.76*** [1.57,1.96]
80+	3.72*** [3.52,3.93]	3.20*** [2.92,3.48]	4.18*** [3.88,4.47]
Sex			
Male^®^	0 [0.00,0.00]		
Female	0.86*** [0.71,1.00]		
Residence			
Rural^®^	0 [0.00,0.00]	0 [0.00,0.00]	0 [0.00,0.00]
Urban	−0.73*** [−0.86,−0.59]	−0.58*** [−0.77,−0.40]	−0.87*** [−1.06,−0.67]
Marital status			
Currently married^®^	0 [0.00,0.00]	0 [0.00,0.00]	0 [0.00,0.00]
Widowed	0.80*** [0.66,0.95]	0.53*** [0.28,0.77]	0.86*** [0.67,1.05]
Others	0.97*** [0.58,1.36]	1.10*** [0.52,1.68]	0.98*** [0.42,1.53]
Education			
No-education^®^	0 [0.00,0.00]	0 [0.00,0.00]	0 [0.00,0.00]
Primary	−0.98*** [−1.13,−0.83]	−0.91*** [−1.11,−0.71]	−1.05*** [−1.27,−0.82]
Secondary and higher secondary	−1.74*** [−1.92,−1.56]	−1.88*** [−2.10,−1.67]	−1.45*** [−1.77,−1.13]
Graduate and above	−2.53*** [−2.84,−2.23]	−2.66*** [−3.01,−2.30]	−2.35*** [−2.97,−1.74]
Caste category			
Scheduled caste^®^	0 [0.00,0.00]	0 [0.00,0.00]	0 [0.00,0.00]
Scheduled tribes	−0.08 [−0.30,0.14]	−0.08 [−0.38,0.22]	−0.14 [−0.46,0.18]
Other backward class	−0.08 [−0.25,0.10]	0.03 [−0.21,0.27]	−0.18 [−0.43,0.07]
Others	0.07 [−0.12,0.25]	−0.03 [−0.29,0.23]	0.15 [−0.12,0.43]
Religion			
Hindu^®^	0 [0.00,0.00]	0 [0.00,0.00]	0 [0.00,0.00]
Muslim	0.13 [−0.05,0.32]	−0.1 [−0.36,0.16]	0.33* [0.06,0.61]
Others	−0.62*** [−0.81,−0.43]	−0.46*** [−0.72,−0.19]	−0.77*** [−1.05,−0.50]
Working status			
Working^®^	0 [0.00,0.00]	0 [0.00,0.00]	0 [0.00,0.00]
Not working	1.38*** [1.24,1.53]	1.63*** [1.44,1.81]	1.18*** [0.94,1.42]
MPCE quantile			
Poorest^®^	0 [0.00,0.00]	0 [0.00,0.00]	0 [0.00,0.00]
Poorer	−0.25** [−0.43,−0.07]	−0.16 [−0.41,0.08]	−0.31* [−0.57,−0.06]
Middle	−0.29** [−0.47,−0.11]	−0.30* [−0.55,−0.05]	−0.26* [−0.52,−0.00]
Richer	−0.32*** [−0.50,−0.13]	−0.34** [−0.60,−0.09]	−0.27* [−0.54,−0.01]
Richest	−0.28** [−0.47,−0.09]	−0.25 [−0.52,0.01]	−0.29* [−0.57,−0.01]
Living Arrangement			
Living with family^®^	0 [0.00,0.00]	0 [0.00,0.00]	0 [0.00,0.00]
Living with others	0.03 [−0.25,0.32]	−0.33 [−0.85,0.19]	0.14 [−0.21,0.48]
Living alone	0.05 [−0.23,0.32]	0.16 [−0.39,0.71]	−0.05 [−0.38,0.28]
Regions			
North^®^	0 [0.00,0.00]	0 [0.00,0.00]	0 [0.00,0.00]
Central	1.25*** [1.04,1.47]	1.15*** [0.86,1.44]	1.35*** [1.04,1.66]
East	1.23*** [1.03,1.42]	1.20*** [0.94,1.47]	1.23*** [0.95,1.52]
West	1.00*** [0.79,1.21]	0.66*** [0.37,0.95]	1.22*** [0.92,1.52]
South	1.02*** [0.83,1.20]	0.80*** [0.54,1.05]	1.17*** [0.90,1.44]
Northeast	0.23* [0.00,0.47]	0.24 [−0.07,0.56]	0.22 [−0.12,0.56]
Ever smoked			
No^®^	0 [0.00,0.00]	0 [0.00,0.00]	0 [0.00,0.00]
Yes	0.14* [0.00,0.27]	0.23** [0.06,0.41]	0.05 [−0.16,0.25]
Ever alcohol			
No^®^	0 [0.00,0.00]	0 [0.00,0.00]	0 [0.00,0.00]
Yes	−0.1 [−0.27,0.07]	−0.26** [−0.45,−0.08]	0.33 [−0.10,0.76]
Physical activity			
No^®^	0 [0.00,0.00]	0 [0.00,0.00]	0 [0.00,0.00]
Yes	−0.70*** [−0.85,−0.56]	−0.70*** [−0.88,−0.52]	−0.74*** [−0.96,−0.51]
Self-rated health			
Good^®^	0 [0.00,0.00]	0 [0.00,0.00]	0 [0.00,0.00]
Poor	3.04*** [2.92,3.16]	2.92*** [2.76,3.09]	3.13*** [2.96,3.30]
Interaction: Chronic Morbidity × Sex			
Female × Single morbidity (ref: male)	0.20 [− 0.07, 0.47]		
Female × Multimorbidity (ref: male)	0.34* [0.05, 0.62]		
Constant	6.48*** [6.35, 6.62]		

The adjusted model includes the following control variables: age group, sex, residence, marital status, education, caste category, religions, working status, MPCE quantile, living arrangement, regions, smoking ever, alcohol ever, physical activity, and self-rated health

^®^- Reference category, β – Beta coefficient, CI - Confidence Interval

^*^p < 0.10

^**^p < 0.05

^***^p < 0.01

Table4 depicts the results from the stepwise regression models examining the association between gender and HRQoL. In the unadjusted model (Model 1), females reported, on average, 2.31 points lower HRQoL scores than men (β = 2.31, 95% CI: 2.18–2.44, p < 0.01). Adjustment for sociodemographic characteristics (Model 2) substantially attenuated this difference to 1.00 points (β = 1.00, 95% CI: 0.86–1.15, p < 0.01), indicating that factors such as age, education, marital status, residence, caste, religion, employment, household economic status, and living arrangement explain more than half of the observed gap. Adding lifestyle and health-related factors (Model 3) produced a modest further reduction, with women still scoring 0.86 points lower than men (β = 0.86, 95% CI: 0.71–1.00, p < 0.01).

**Table 4:** Regression results for the associations of HRQoL with gender

Table 4: Regression results for the associations of HRQoL with gender						
HRQoL score	Model-1 (Unadjusted)		Model-2 (semi-adjusted)		Model-3 (full-adjusted)	
	β [95% CI]		β [95% CI]		β [95% CI]	
Sex						
Male^®^	0	[0.00,0.00]	0	[0.00,0.00]	0	[0.00,0.00]
Female	2.31***	[2.18,2.44]	1.00***	[0.86,1.15]	0.86***	[0.71,1.00]

The semi-adjusted model includes the following control variables: age group, sex, residence, marital status, education, caste category, religions, working status, MPCE quantile, living arrangement, regions, and full-adjusted model include the following control variables: age group, sex, residence, marital status, education, caste category, religions, working status, MPCE quantile, living arrangement, regions smoking ever, alcohol ever, physical activity, and self-rated health

^®^- Reference category, β – Beta coefficient, CI - Confidence Interval

^*^p < 0.10

^**^p < 0.05

^***^p < 0.01

Table 5 depicts the association of chronic disease with HRQoL among older adults. The results were presented in three models for males and females, with a β coefficient with 95% CI. The analysis in model-1 for total indicated that the adjusted β coefficients were 0.52 (β: 0.52; 95%CI: 0.39,0.65; P: <0.001) for Hypertension, 0.19 (β: 0.19; 95%CI: 0.02,0.36; P: <0.05) for Diabetes, 1.18 (β: 1.18; 95%CI: 0.53,1.84; P: <0.05) for Cancer, 0.62 (β: 0.62; 95%CI: 0.40,0.83; P: <0.001) for Lung disease, 0.76 (β: 0.76; 95%CI: 0.50,1.02; P: <0.001) for Heart disease, 3.77 (β: 3.77; 95%CI: 3.40,4.14; P: <0.001) for Stroke, 2.46 (β: 2.46; 95%CI: 2.09,2.82; P: <0.001) for Neurological, and 2.07 (β: 2.07; 95%CI: 1.92,2.22; P: <0.001) for Bone and joint, concerning no disease reference. This result indicates that chronic diseases such as Stroke, Neurological, and Bone and joint were significantly associated with lower HRQoL scores than other diseases. Similar results show an association of chronic disease in HRQoL males and females in models 2 and 3. Interaction tests (Table 5 footnote) revealed that the association between high cholesterol and HRQoL significantly differed by gender (β for female interaction = 0.73, 95%CI: 0.04, 1.43). Men with high cholesterol exhibited significantly lower HRQoL (β = −0.44, 95%CI: −0.88,−0.00), while women showed no significant association (β = 0.22, 95%CI: −0.21,0.66). For other chronic conditions, such as stroke, neurological disorders, and bone/joint diseases, the direction and magnitude of associations with HRQoL were similar across genders, though point estimates were generally higher in women. However, these gender differences were not statistically significant.

**Table 5:** Adjusted regression results for associations of HRQoL with chronic conditions

HRQoL Score	Model-1 (Total)	Model-2 (Male)	Model-3 (Female)
	β [95% CI]	β [95% CI]	β [95% CI]
Hypertension			
No^®^	0 [0.00,0.00]	0 [0.00,0.00]	0 [0.00,0.00]
Yes	0.52*** [0.39,0.65]	0.56*** [0.37,0.74]	0.48*** [0.30,0.66]
Diabetes			
No^®^	0 [0.00,0.00]	0 [0.00,0.00]	0 [0.00,0.00]
Yes	0.19* [0.02,0.36]	0.1 [−0.12,0.33]	0.27* [0.03,0.52]
Cancer			
No^®^	0 [0.00,0.00]	0 [0.00,0.00]	0 [0.00,0.00]
Yes	1.18*** [0.53,1.84]	0.62 [−0.32,1.57]	1.57*** [0.66,2.48]
Lung disease			
No^®^	0 [0.00,0.00]	0 [0.00,0.00]	0 [0.00,0.00]
Yes	0.62*** [0.40,0.83]	0.47** [0.19,0.76]	0.77*** [0.44,1.10]
Heart disease			
No^®^	0 [0.00,0.00]	0 [0.00,0.00]	0 [0.00,0.00]
Yes	0.76*** [0.50,1.02]	0.81*** [0.47,1.15]	0.74*** [0.33,1.16]
Stroke			
No^®^	0 [0.00,0.00]	0 [0.00,0.00]	0 [0.00,0.00]
Yes	3.77*** [3.40,4.14]	4.02*** [3.56,4.48]	3.43*** [2.83,4.02]
Neurological			
No^®^	0 [0.00,0.00]	0 [0.00,0.00]	0 [0.00,0.00]
Yes	2.46*** [2.09,2.82]	2.44*** [1.94,2.93]	2.48*** [1.95,3.02]
High cholesterol			
No^®^	0 [0.00,0.00]	0 [0.00,0.00]	0 [0.00,0.00]
Yes	−0.06 [−0.37,0.25]	−0.44* [−0.88,−0.00]	0.22 [−0.21,0.66]
Bone/joint			
No^®^	0 [0.00,0.00]	0 [0.00,0.00]	0 [0.00,0.00]
Yes	2.07*** [1.92,2.22]	2.00*** [1.78,2.22]	2.09*** [1.88,2.30]

The adjusted model for each morbidity in all models includes the following control variables: age group, sex, residence, marital status, education, caste category, religions, working status, MPCE quantile, living arrangement, regions, smoking ever, alcohol ever, physical activity, and self-rated health. Interaction effects between gender (reference: male) and individual chronic conditions on HRQoL were tested. Coefficients [95% CI] for females: Hypertension = 0.14 (− 0.14, 0.41); Diabetes = 0.30 (− 0.97, 0.66); Cancer = 0.24 (− 1.31, 1.78); Chronic lung disease = 0.11 (− 0.39, 0.61); heart disease = 0.30 (− 0.31, 0.90); Stroke = − 0.40 (− 1.32, 0.39); High cholesterol = 0.73 (0.04, 1.43); Bone/joint disorders = − 0.03 (− 0.38, 0.31)

^®^- Reference category, β – Beta coefficient, CI - Confidence Interval

^*^p < 0.10

^**^p < 0.05

^***^p < 0.01

## Discussion

Multimorbidity was more prevalent among the older population, negatively impacting people’s quality of life and overall well-being. The health-related quality of life (HRQoL) reflects the overall health and well-being, including physical, mental, and social dimensions of well-being, and the studies show that multimorbidity conditions significantly negatively impact HRQoL. However, there was a drought in literature in the Indian context, especially among older populations, on how multimorbidity is associated with HRQoL and how gender plays a role. With this gap, this study examined the HRQoL and its association with multimorbidity among older adults in India.

Our study has found that older individuals with a morbidity condition had a significantly lower level of HRQoL. Previous studies have demonstrated an inverse relationship between chronic conditions and HRQoL [2, 12, 22, 26, 39]. HRQoL was a measure of health status commonly used to assess the medical effectiveness of interventions and to support public health planning, with the EQ-5D serving as a generic tool for comparing populations with different conditions. In this study, we utilized proxy EQ-5D scores for the analysis. Our findings indicate that HRQoL was not only lower among individuals with multimorbidity but also among those with lower socioeconomic status, including females, rural residents, individuals with limited education, those in the poorest wealth quintile, and those in poor health. These results align with previous research highlighting the impact of chronic conditions and socioeconomic factors on HRQoL [23, 39].

Globally, the study shows that chronic conditions lead to diminished HRQoL, reduced physical functioning, and a higher risk of depression and anxiety [40]. A study in Korea shows that older women had a negative association of multimorbidity with HRQoL [41]. Additionally, a study in India by Pati et al., 2019 found a significantly high impairment of physical and mental HRQoL in Patients with Multimorbidity above 50 years, and different combinations of chronic conditions appear to impact HRQoL differently [32].

Furthermore, the our study found that both single and multimorbidity prevalence were higher among females than among males. Additionally, females reported significantly lower HRQoL scores than their male counterparts. HRQoL was strongly associated with chronic conditions; individuals with multimorbidity had substantially lower HRQoL scores than those with only one or no chronic condition. Specifically, chronic diseases such as stroke, neurological disorders, heart diseases, and bone and joint conditions were significantly linked to lower HRQoL levels. This association also varied by gender. The interaction tests confirmed that multimorbidity (aggregated conditions) had a stronger association with poorer HRQoL in females. For individual chronic conditions, associations were directionally consistent across genders but not significantly stronger in females, except for high cholesterol, which uniquely affected males. This suggests that the overall burden of multimorbidity, rather than specific conditions, disproportionately impacts HRQoL among females.

Previous results from studies by Sharma et al. (2023), Arokiasamy et al. (2015), and Pati et al. (2019) in India also show that the prevalence of multimorbidity was higher among females than males [31, 32, 42]. The primary reason for the higher disease burden among women than men was their higher life expectancy. As women age, they are more likely to experience chronic morbidity and other age-related difficulties. Studies have shown that while women live longer than men, they spend more years in poor health, with conditions such as musculoskeletal disorders, mental health issues, and frailty becoming more prevalent with age [10, 12, 43–45]. This phenomenon, known as the health-survival paradox, underscores the complex interplay between longevity and health outcomes [46].

The higher prevalence of chronic morbidity among women compared to men can be attributed to gender inequalities in resource allocation, including disparities in income, education, and healthcare. Chauhan et al. (2022) found that women, often disadvantaged in these areas, were more likely to experience multimorbidity. Their study highlighted that individuals with higher education levels, urban residents, and those in higher wealth quintiles had significantly higher risks of multimorbidity, factors that disproportionately affect women in India [47]. A study conducted in Iran on elderly individuals found that women had significantly lower HRQoL scores in all subscales compared with men after adjusting for various factors [34].

The stepwise regression results highlight that socio-economic factors played a substantial role in explaining the gender gap in HRQoL. In the unadjusted model, women had significantly higher HRQoL scores (β = 2.31) compared to men. After accounting for demographic and socio-economic characteristics such as age, education, marital status, residence, caste, religion, employment, household economic status, and living arrangement in the semi-adjusted model, the gender difference reduced to β = 1.00, which is representing approximately 57% decline. However, lifestyle and health-related factors, such as smoking, alcohol consumption, physical activity, and self-rated health, were additionally considered in the full-adjusted model, the gender gap further declined modestly to β = 0.86, an additional 14% reduction. Overall, about 63% of the initial gender difference in HRQoL was explained by observed covariates, while approximately 37% of the disparity remained unexplained.

Despite adjusting for a wide range of sociodemographic, economic and health characteristics, our findings reveal persistent gender differences in both multimorbidity prevalence and HRQoL among older adults. This suggests that these disparities are not fully explained by observable background factors. Biological sex differences may partially account for these variations, as women tend to live longer and may accumulate more chronic conditions over time [48]. Additionally, gendered patterns in health reporting have been observed, with women more likely to report health problems and limitations, potentially leading to higher observed morbidity and lower HRQoL [49, 50]. Moreover, older women in India often experience lifelong structural disadvantages, including limited access to healthcare, education, and decision-making, which may exacerbate their health conditions in later life [51].

Lower education, poorest wealth, and poor health are also inversely related to lower HRQoL in our study. The management of multimorbidity is complex, caused primarily by the challenges of specialized healthcare, which results in fragmented care, polypharmacy, multiple treatment burdens, mental health problems, and increased healthcare utilization that strains available resources [12, 21, 52–54], which O’Brien described as an “endless struggle” for comorbidity patients [55]. When dealing with increased threats from multimorbidity, two priorities need to be addressed by government and professional entities.

Chronic condition management should target functions and abilities necessary to manage daily household and social activities, not only the disease itself [56]. Revisions to clinical guidelines and research protocols are required because they focus only on care programs for single diseases. A more integrated and person-centered approach is needed, as the cumulative application of individual disease-specific recommendations can be impractical and even conflicting for patients with multiple conditions. The traditional clinical guidelines might be appropriate for patients with a single condition, but the sum of clinical recommendations from various sources suggests different approaches for multimorbidity [32, 57].

Our study is among the first to assess the relationship between multimorbidity and HRQoL in an Indian primary care context. We found an inverse relationship between the number of chronic conditions and HRQoL, for both physical and mental domains. Notably, the severity of conditions appears to be an even stronger predictor of diminished well-being than the mere count of chronic diseases. Therefore, a comprehensive assessment of multimorbidity should incorporate both the number and severity of conditions to better inform clinical decision-making and public health planning.

### Limitations and future research directions

This study provides important insights into the relationship between multimorbidity and health-related quality of life (HRQoL) among older adults in India; however, certain limitations must be acknowledged. The measurement of HRQoL was based on proxy EQ-5D indicators using binary responses, which may not fully reflect the range of quality-of-life experiences and limit comparability with studies using the standard EQ-5D-5 L tool. Additionally, multimorbidity was defined using nine physician-diagnosed conditions, which, while consistent with prior LASI studies, may underestimate true prevalence compared to definitions using a broader set of conditions [58]. Moreover, the cross-sectional nature of the study restricts the ability to draw causal inferences. Despite these limitations, the study is strengthened by its use of nationally representative data, enhancing its generalizability to the broader older population in India. Future research should adopt longitudinal designs to examine causal pathways, incorporate all available chronic conditions in LASI to improve consistency, and explore disease-specific patterns and severity of multimorbidity. Employing validated instruments such as EQ-5D-5 L can also offer a more accurate assessment of HRQoL and guide targeted, evidence-based interventions for healthy ageing.

## Conclusions

In order to achieve the goals of healthy ageing and well-being in India, this study highlights the urgent need to address the health-related quality of life (HRQoL) of older adults, particularly those living with multimorbidity. The results indicate that increasing age, female gender, widowed status, low educational attainment, poor economic status, and self-rated poor health are all significantly associated with lower HRQoL, with women bearing a disproportionately higher burden. These findings call for targeted public health interventions such as integrated chronic disease management, gender-sensitive care strategies, and improved access to geriatric services, especially in underserved areas. Strengthening primary healthcare systems to offer coordinated, person-centered care for older adults could play a vital role in improving their overall quality of life and reducing health inequalities. Policymakers should consider these insights when designing and implementing national ageing and non-communicable disease programs.

## Data Availability

The study utilizes a secondary source of data from the Longitudinal Ageing Study in India (LASI) that is freely available in the public domain through a request form (LASI Wave 1 Data Request form (https://www.iipsdata.ac.in/datacatalog_detail/5).

## Abbreviations

CES-D: Center for Epidemiological Studies-Depression scale
CI: Confidence Interval
CVDs: Cardiovascular Diseases
EQ-5D: EuroQol Five-Dimension
HRQoL: Health-Related Quality of Life
LASI: Longitudinal Ageing Study in India
MPCE: Monthly Per Capita Consumption Expenditure
SD: Standard Deviation
WHO: World Health Organization
